# Association between promoter methylation of *DAPK* gene and HNSCC: A meta-analysis

**DOI:** 10.1371/journal.pone.0173194

**Published:** 2017-03-01

**Authors:** Fucheng Cai, Xiyue Xiao, Xun Niu, Yi Zhong

**Affiliations:** 1 Department of Pediatrics, Union Hospital, Tongji Medical College, Huazhong University of Science and Technology, Wuhan, China; 2 Department of Obstetrics and Gynecology, Union Hospital, Tongji Medical College, Huazhong University of Science and Technology, Wuhan, China; 3 Department of Otorhinolaryngology, Union Hospital, Tongji Medical College, Huazhong University of Science and Technology, Wuhan, China; University of Cincinnati College of Medicine, UNITED STATES

## Abstract

**Background:**

The death-associated protein kinase (*DAPK*) is a tumor suppressor gene, which is a mediator of cell death of INF-γ–induced apoptosis. Aberrant methylation of *DAPK* promoter has been reported in patients with head and neck squamous cell carcinoma (HNSCC). However, the results of these studies are inconsistent. Hence, the present study aimed to evaluate the association between the promoter methylation of *DAPK* gene and HNSCC.

**Methods:**

Relevant studies were systematically searched in PubMed, Web of Science, Ovid, and Embase. The association between *DAPK* promoter methylation and HNSCC was assessed by odds ratio (ORs) and 95% confidence intervals (CI). To evaluate the potential sources of heterogeneity, we conducted the meta-regression analysis and subgroup analysis.

**Results:**

Eighteen studies were finally included in the meta-analysis. The frequency of *DAPK* promoter methylation in patients with HNSCC was 4.09-fold higher than the non-cancerous controls (OR = 3.96, 95%CI = 2.26–6.95). A significant association between *DAPK* promoter methylation and HNSCC was found among the Asian region and the Non-Asia region (Asian region, OR = 4.43, 95% CI = 2.29–8.58; Non-Asia region, OR = 3.39, 95% CI = 1.18–9.78). In the control source, the significant association between *DAPK* promoter methylation and HNSCC was seen among the autologous group and the heterogeneous group (autologous group, OR = 2.71, 95% CI = 1.49–4.93; heterogeneous group, OR = 9.50, 95% CI = 2.98–30.27). *DAPK* promoter methylation was significantly correlated with alcohol status (OR = 1.85, 95% CI = 1.07–3.21).

**Conclusion:**

The results of this meta-analysis suggested that aberrant methylation of *DAPK* promoter was associated with HNSCC.

## Introduction

Head and neck squamous cell carcinoma(HNSCC)is the sixth most common cancer worldwide [1]. More than 500,000new HNSCC cases are diagnosed each year, which include two-thirds of the patients diagnosed with advanced stage, lymph node metastasis [2]. Moreover, the five-year survival of patients with HNSCC remains about 40–50% [2].The molecular mechanisms associated with the pathogenesis of HNSCC comprise of a variety of genetic alterations such as mutations and epigenetic modifications, including methylation of CpG islands. In addition, the epigenetic modification resulting in the alteration of expression of tumor-related genes is considered crucial in the development of HNSCC [3,4].

The promoter methylation of the tumor suppressor gene (TSG) leads to gene inactivation, which reduces or inhibits the function of the tumor suppressor. Hypermethylation of the tumor suppressor gene occurs in cancer development for many types of cancers including HNSCC. The death-associated protein kinase (*DAPK*) is a tumor suppressor gene, which is a mediator of cell death of INF-γ–induced apoptosis [5–7]. The decreased expression of *DAPK* is associated with the methylation of gene promoter [8,9]. The methylation of *DAPK* promoter has been found to be an important epigenetic modification in several types of cancers [10–12].

Aberrant methylation of *DAPK* promoter has been reported in patients with HNSCC. However, the results are inconsistent. There are significant differences in the frequency of *DAPK* promoter methylationin patients with HNSCC. Moreover, whether the methylation frequency of *DAPK* promoter is correlated with clinicopathological features (sex, smoking status, alcohol status and lymph node invasion) in HNSCC patients remains unclear. Thus, we performed the meta-analysis to investigate the relationship between the methylation status of *DAPK* promoter and HNSCC, as well as the relationship between *DAPK* promoter methylation and clinicopathological features of HNSCC.

## Materials and methods

The meta-analysis was performed according to the latest meta-analysis guidelines (PRISMA) [13].

### Search strategy

Systematic review of relevant literature was conducted using PubMed, Web of Science, Ovid, and Embase databases from January 1, 1968, to June 30, 2016. The keywords used for the literature search were: (*DAPK* methylation) and (head and neck or oral or tonsil ororopharyngeal or laryngeal or oropharynx) and (squamous cell carcinoma or cancer).

### Inclusion and exclusion criteria of literature

The studies were included if they satisfied the following inclusion criteria: (1) investigated the correlation between *DAPK* promoter methylation and HNSCC or investigated the correlation between *DAPK* promoter methylation and clinicopathological features,(2) specimens of case group (HNSCC) were limited to tissues, (3) the *DAPK* promoter methylation frequency and sample size provided in the case and the control groups.

Only studies written in English were included for review. In addition, case reports, abstracts, and letters to the editor were eliminated.

### Data extraction and quality assessment

The relevant data from the eligible studies were independently retrieved by two authors (Fucheng Cai and Yi Zhong). The relevant data include the name of the first author, year of publication, region of study subjects, age of patients, methylation detection method, source of control, type of samples in the control group, number of people with *DAPK* methylation in case and control groups, and sample size of case and control groups. Moreover, we also extracted the number of individuals with *DAPK* methylation in clinical features’ subgroups in the studies investigating the correlation between *DAPK* promoter methylation and clinical characteristics of HNSCC. The third reviewer (Xiyue Xiao) independently reviewed the relevant data extracted from the eligible studies.

### Statistical analysis

The strength of the association between *DAPK* promoter methylation and HNSCC was evaluated by odds ratio (OR) with 95% confidence intervals (CIs).The degree of association between *DAPK* promoter methylation and clinicopathological features was also evaluated by OR with 95%CI.The heterogeneity among the included studies was estimated by the Cochran Q test and *I*^*2*^ statistics [14].The random-effects model was used to compute the pooled ORs when the heterogeneity was considered significant (*P*<0.05 for the Q statistic). In the case of a different scenario, a fixed-effects model was applied to compute the pooled ORs. To explore the potential source of heterogeneity among the included studies, meta-regression analyses, and subgroup analyses were conducted. A sensitivity analysis was employed to assess the influence of each study excluded in the combined OR. The publication bias was assessed by the Begg’s funnel plot [15]and Egger’s test [16]. The reported P values were two-sided for all the analyses. 0.5 is added as a default to all 0 counts when the 2×2 table for the individual studies contains cells with 0 counts in the Meta package. All statistical tests were performed using the Meta package in R (version 3.2.3; http://www.r-project.org/).

## Results

### Identification of studies and study characteristics

**Fig 1:**
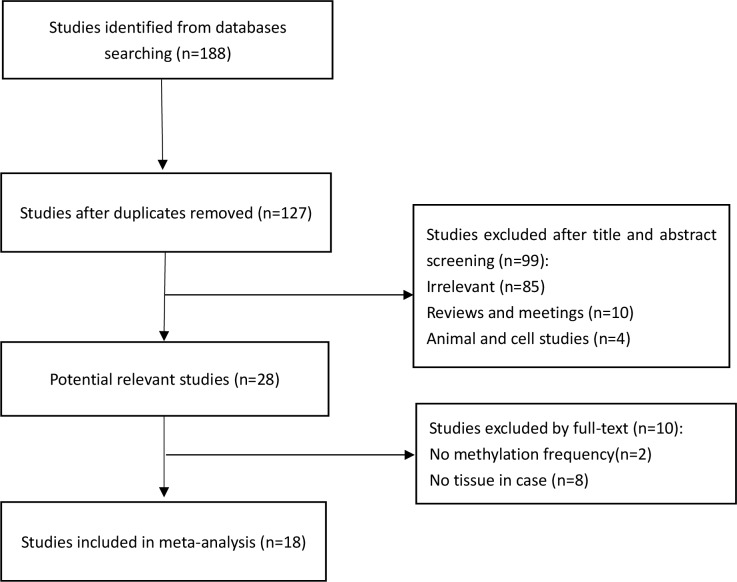
Flow chart of studies included in the meta-analysis.

A total of 188 studies were initially identified by literature search. The duplicates and non-relevant studies (reviews and animal and cell studies) were excluded by considering the title and abstract of the studies. 28 articles with potentially relevant studies were further identified by examining the full text. Finally, 18 studies were included in the meta-analysis after excluding studies without methylation frequency and tissues in the case group. The detailed study selection process is illustrated in Fig 1.

Out of the 18 studies included, 15 studies with 818 cases and 852 controls were combined to calculate the pooled OR between *DAPK* promoter methylation and HNSCC. The 15 studies encompassed the publication years from 2002–2015. The methylation detection methods consisted of the methylation-specific polymerase chain reaction (MSP), real-time quantitative MSP (QMSP), and bisulfite sequencing PCR (BSP). Among the 15 included studies, 10 studies used MSP, 4 studies used QMSP and 1 study used BSP to explore *DAPK* promoter methylation in HNSCC and corresponding control. Eight studies were of Asian subjects and seven studies were of non-Asian subjects. The sample of controls consisted of tissue, blood, saliva, and buccal scrapings. The control source contained autologous and heterogeneous controls. The detail study characteristics were summarized in Table 1.

**Table 1:** Characteristics of studies included in the meta-analysis of *DAPK* promoter methylation and HNSCC.

author	year	region	age (case, years)	case		control		method^#^	control source*	control sample
				M	U	M	U			
Arantes, L. M.[21]	2015	Brazil	median = 54.5;range:41–78	32	8	8	32	QMSP	H	saliva
Choudhury, J. H.[22]	2015	India	range:23–86	21	50	5	40	MSP	A	tissue
Rettori, M. M.[23]	2013	Brazil	median = 59;range:20–90	35	33	1	38	QMSP	H	saliva
Li, C.[8]	2013	China	median = 55;range:40–72	30	23	0	23	MSP	H	tissue
Liu, Y.[24]	2012	China	mean = 55.0; sd: 13.5	15	17	15	62	QMSP	H	tissue
						16	61			blood
						2	75			saliva
Paluszczak, J.[25]	2011	Poland	mean = 58.3;range:41–75	31	10	32	9	MSP	A	tissue
Wong, Y.K.[26]	2011	Taiwan	mean = 51.7;range:26–77	29	35	26	38	MSP	A	tissue
						0	20		H	tissue
Laytragoon-Lewin, N.[27]	2010	Sweden	median = 62;range:42–101	7	11	2	16	MSP	A	tissue
Su, P. F.[28]	2010	Taiwan	mean = 54.94;range:37–82	13	18	5	26	QMSP	A	tissue
						0	12		H	buccal scrapings
Steinmann, K.[29]	2009	Germany	mean = 57;range:41–71	36	18	8	15	MSP	A	tissue
De Schutter, H.[30]	2009	Belgium	mean = 59;range:43–76	4	40	2	3	MSP	H	tissue
Righini, C. A.[31]	2007	French	median = 57;range:33–74	19	71	1	29	MSP	A	tissue
						9	51		A	saliva
						0	30		H	saliva
Kong, W. J.[32]	2005	China	mean = 53.3;range:32–78	39	19	6	52	MSP	A	tissue
Kulkarni, V.[33]	2004	India	median = 50;range:25–71	41	19	36	24	MSP	A	tissue
						0	20		H	buccal scrapings
Ogi, K.[17]	2002	Japan	NA	7	89	0	2	BSR	H	tissue

M: *DAPK* promoter methylated, U: *DAPK* promoter unmethylated

#: MSP: methylation-specific polymerase chain reaction, QMSP: real-time quantitative MSP, BSP: Bisulfite sequencing PCR

*: A: Autologous control, H: Heterogeneous control

Among the 18 included studies, seven studies were combined to estimate the pooled OR between *DAPK* promoter methylation and clinicopathological features of HNSCC from the 18 included studies. The clinicopathological features included sex, smoking status, alcohol status, and lymph node invasion. The detailed characteristics of the study were summarized in Table 2.

**Table 2:** Characteristics of studies included in themeta-analysis of *DAPK* promoter methylation and clinicopathological features.

Author	Year	Region	Method^#^	Sex		Smoking		Alcohol		N_stage*	
				Male (M/U)	Female (M/U)	Smoker (M/U)	Non-smoker (M/U)	Yes (M/U)	No (M/U)	N0 (M/U)	N+ (M/U)
Misawa, K.[34]	2016	Japan	QMSP	54/55	17/7	53/42	18/20	51/36	20/26	31/28	31/28
Arantes, L. M.[21]	2015	Brazil	QMSP	29/7	3/1	26/3	6/5	22/4	10/4	9/2	23/6
Pierini, S.[35]	2014	Bulgaria	MS-HRM	37/54	4/2	28/44	13/12			29/43	12/13
Wong, Y.K.[26]	2011	Taiwan	MSP	25/33	4/2	20/30	7/7	18/22	5/9		
Su, P. F.[28]	2010	Taiwan	QMSP	12/17	2/2	11/13	3/5	8/9	3/8	5/6	9/13
Supic, G.[36]	2009	Serbia	MSP	25/39	3/10	22/39	6/10			4/12	24/37
Kulkarni, V.[33]	2004	India	MSP	29/15	12/4					22/10	19/9

M: *DAPK* promoter methylated, U: *DAPK* promoter unmethylated

#: MSP: methylation-specific polymerase chain reaction, QMSP: real-time quantitative MSP, MS_HRM: Methylation-sensitive high resolution melting

*: N_stage: lymph node invasion

### Association between *DAPK* promoter methylation and HNSCC

**Fig 2:**
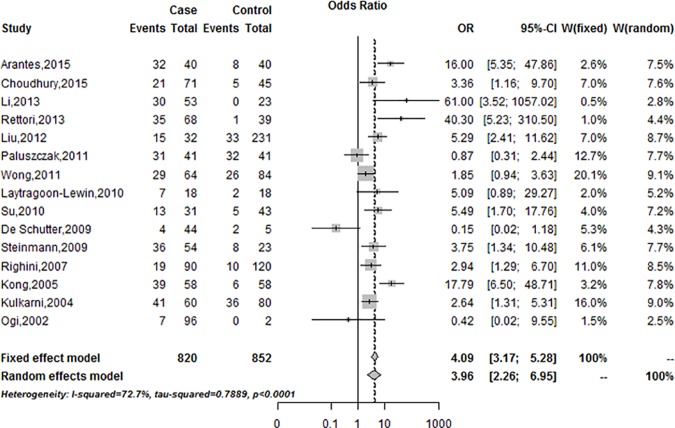
Forest plots of *DAPK* promoter methylation associated with HNSCC.

In the meta-analysis, the heterogeneity among the included studies was significant for Q test (P<0.001). Thus, the random-effect model was employed to evaluate the summary of ORs. In the random-effect model, we found that *DAPK* promoter methylation was significantly associated with HNSCC (pooled OR = 3.96,95%CI = 2.26–6.95) (Fig 2).

### Association between *DAPK* promoter methylation with clinicopathological features

**Fig 3:**
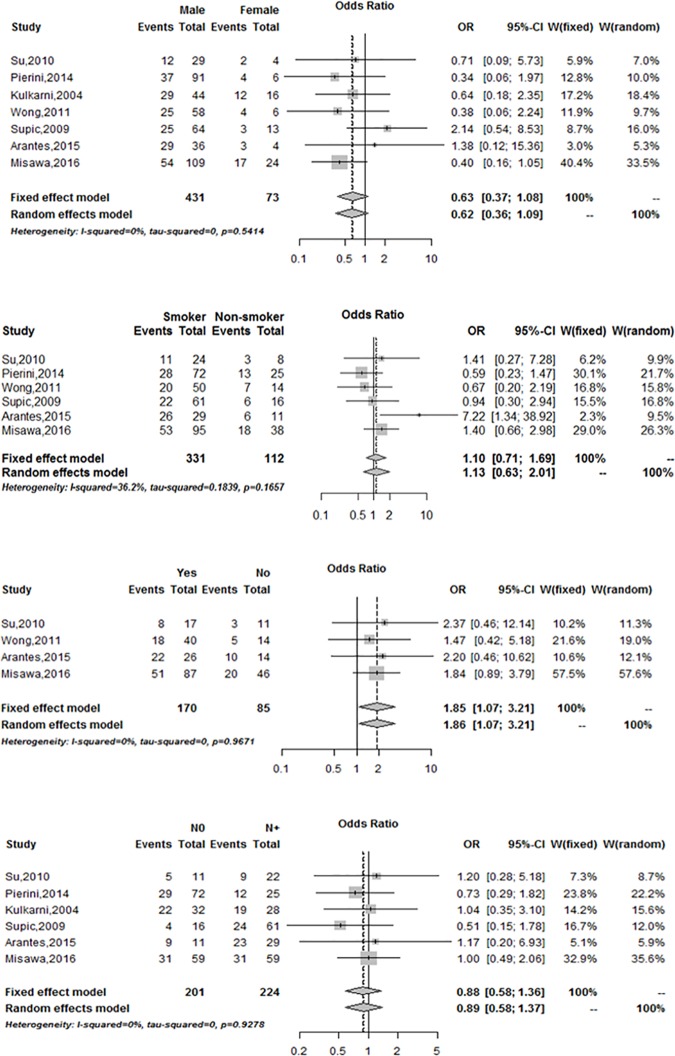
Forest plots of *DAPK* promoter methylation associated with clinicopathological features A: Forest plots of *DAPK* promoter methylation associated with sex B: Forest plots of *DAPK* promoter methylation associated with smoking status C: Forest plots of *DAPK* promoter methylation associated with alcohol status D: Forest plots of *DAPK* promoter methylation associated with lymph node invasion.

The meta-analysis result suggested that the frequency of DAPK promoter methylation in patients with HNSCC was significantly higher than the corresponding controls (Fig 2).Therefore, we also assessed the association between *DAPK* promoter methylation and the clinicopathological features. Among the included studies, the smoking group was divided into three groups (Current, Former, and Never) in three studies. The smoking group in the three studies was divided into two groups (Smoker and Non-smoker). To pool the data, the Current group was classified as Smoker group, and the Former and Never groups were classified as Non-smoker group. In the meta-analysis, *DAPK* promoter methylation was not significantly correlated with sex, smoking status, and lymph node invasion (Fig 3A, 3B and 3D). However, the meta-analysis found that *DAPK* promoter methylation was significantly correlated with the alcohol status (OR = 1.85, 95% CI = 1.07–3.21) (Fig 3C).

### Meta-regression analysis and subgroup analysis

The meta-regression analysis was used to explore the potential sources of heterogeneity among the included studies. We found that the possible source of heterogeneity was the method (*P* = 0.04) according to the meta-regression analysis (Table 3). To further assess the potential sources, we conducted the subgroup analysis according to the region, methylation detection method, control source, control sample type, and sample size of the case group.

**Table 3:** Meta-regression analysisof *DAPK* promoter methylation and HNSCC.

		95%CI		
Heterogeneity sources	Coefficient	Lower	Upper	*P*
Publication year	0.062	-0.115	0.239	0.495
Region	-0.881	-2.125	0.363	0.165
Method	-1.825	-3.590	-0.060	0.043
Case sample size	-0.767	-2.113	0.580	0.265
Control source	1.256	-0.417	2.929	0.141
Control sample	-1.474	-3.033	0.086	0.064

With respect to the subgroups categorized by the region, significant association between *DAPK* promoter methylation and HNSCC was found among the Asian region and the Non-Asia region in the random-effect model (Asian region, OR = 4.43, 95% CI = 2.29–8.58; Non-Asia region, OR = 3.39, 95% CI = 1.18–9.78).The heterogeneity did not decrease remarkably among the region-based subgroup. In the methylation detection method group, Ogi et al.[17] used bisulfite-PCR (BSP) to detect methylation and was classified as the MSP group.

The significant association between *DAPK* promoter methylation and HNSCC was displayed among the MSP in the random-effect model and the QMSP in the fixed-effect model (MSP, OR = 2.97, 95% CI = 1.55–5.70; QMSP, OR = 8.84, 95% CI = 5.22–14.99). In the control source, the significant association between *DAPK* promoter methylation and HNSCC was seen among the autologous group and the heterogeneous group in the random-effect model (autologous group, OR = 2.71, 95% CI = 1.49–4.93; heterogeneous group, OR = 9.50, 95% CI = 2.98–30.27). With the control sample type, a significant association between *DAPK* promoter methylation and HNSCC was found among the tissue group and the non-tissue group in the random-effect model (tissue group, OR = 3.95, 95% CI = 1.89–8.25; non-tissue group, OR = 5.30, 95% CI = 2.17–12.93). With the sample size in the cases, significant association between *DAPK* promoter methylation and HNSCC was found among the <60group in random-effect model and the ≥60 group in the fixed-effect model (<60 group, OR = 4.64, 95% CI = 1.94–11.06; ≥60 group, OR = 3.12, 95% CI = 2.17–4.49). The subgroup analysis of *DAPK* promoter methylation associated with HNSCC was summarized in Table 4.

**Table 4:** Summary of the subgroup analysisin the meta-analysis of *DAPK* promoter methylation and HNSCC.

Group	Case		Control		Fixed-effects model	Random-effects model	Heterogeneity		
	M+	N	M+	N	OR (95%CI)	OR (95%CI)	*I*^*2*^ (%)	*P*	τ^2^
Total	359	820	174	852	4.09 (3.17–5.28)	3.96 (2.26–6.95)	72.7	<0.001	0.79
Region									
Asia	195	465	111	566	4.21 (3.04–5.84)	4.43 (2.29–8.58)	67.4	0.003	0.53
Non-asia	164	355	63	286	3.91 (2.60–5.90)	3.39 (1.18–9.78)	79.8	<0.001	1.53
Method									
MSP	257	553	127	497	3.18 (2.37–4.28)	2.97 (1.55–5.70)	72.8	<0.001	0.72
QMSP	102	267	47	355	8.84 (5.22–14.99)	7.73 (3.09–19.36)	56.4	0.06	0.56
Control source									
Autologous	236	487	130	430	2.49 (1.84–3.36)	2.71 (1.49–4.93)	70.3	0.001	0.56
Heterogeneous	225	578	44	442	11.46 (6.85–19.18)	9.50 (2.98–30.27)	70.8	<0.001	2.12
Control sample type^$^									
Tissue	292	712	138	497	3.41 (2.55–4.54)	3.95 (1.89–8.25)	75.4	<0.001	1.14
Non-tissue	155	321	36	355	6.31 (4.09–9.73)	5.30 (2.17–12.93)	69.8	0.002	1.03
Case sample size									
<60	207	371	96	482	5.35 (3.72–7.71)	4.64 (1.94–11.06)	77.8	<0.001	1.27
≥60	152	449	78	370	3.12 (2.17–4.49)	2.94 (1.60–5.38)	50.9	0.070	0.26

M+: *DAPK* promoter methylated

N: total number

### Sensitivity analysis

**Fig 4:**
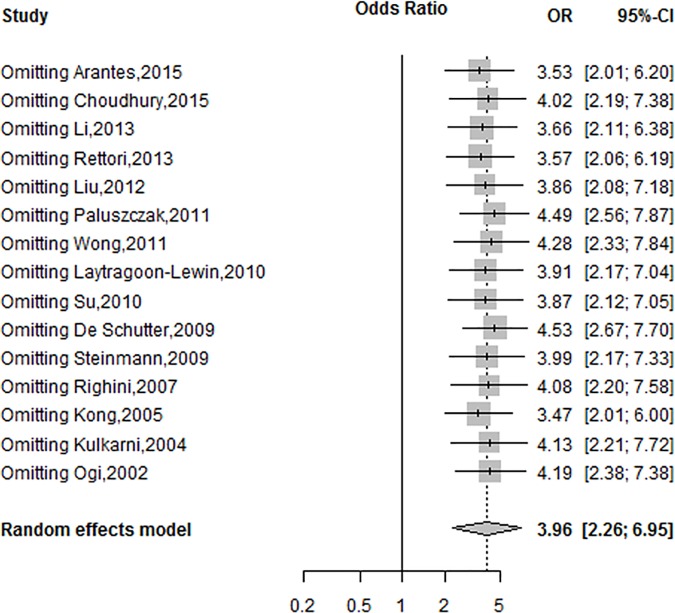
Sensitivity analysis of *DAPK* promoter methylation and HNSCC by the random-effects method.

The sensitivity analysis was performed to evaluate the stability of the conclusions according to the leave-one-out method by excluding one study. The pooled OR was changed from 3.47 (95%CI = 2.01–6.00) to 4.53(95%CI = 2.67–7.70) under the random-effect model, which confirms the stability of the results (Fig 4). Therefore, the result of the meta-analysis was stable and reliable.

### Publication bias

**Fig 5:**
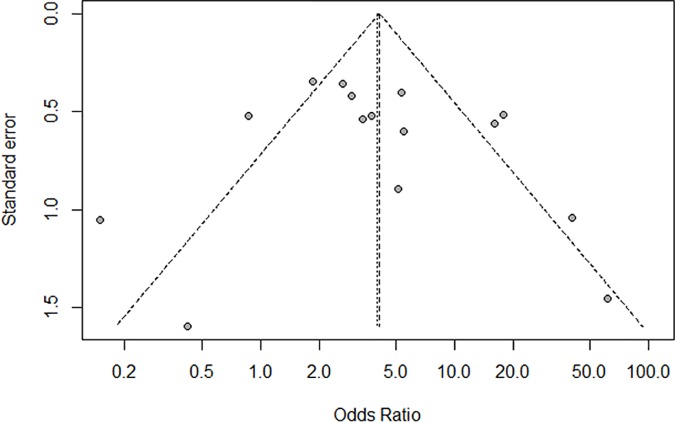
Begg’s funnel plot of *DAPK* promoter methylation associated with HNSCC.

Publication bias of the included studies was assessed through the Begg’s funnel plot and Egger’s test. The shape of the Begg’s funnel plot did not reveal any potential asymmetry (Fig 5). The publication bias detected by Egger’s test was not significant (*P* = 0.55).

## Discussion

Hypermethylation of the promoter of the tumor suppressor gene (TSG) resulted in silencing the expression of TSGs in carcinogenesis of the tumor. Death-associated protein kinase (*DAPK*), a tumor suppressor gene, could mediate cell death in INF-γ–induced apoptosis, whereas inactivated *DAPK*, could lead to the pathogenesis and metastasis of the tumor [18]. The loss of expression of *DAPK* mainly induced by methylation of its promoter plays a crucial role in the carcinogenesis of the tumor [19].

The present meta-analysis including 15 studies was performed to quantitatively assess the strength of association of DAPK promoter methylation and HNSCC. The overall frequency of DAPK promoter methylation in patients with HNSCC was 43.64% and 20.42% in the control population. The results of the meta-analysis suggested that individuals with hypermethylation of DAPK promoter are associated with HNSCC (pooled OR = 3.96, 95%CI = 2.26–6.95).

A significant heterogeneity between the studies was found by Q-test in the meta-analysis. The subgroup analysis was conducted to explore the potential heterogeneity among the included studies in our meta-analysis; a significant association between *DAPK* methylation and HNSCC was found in all the subgroup (Table 4). In the methylation detection method group, a significant association between *DAPK* promoter methylation and HNSCC was observed among the MSP in the random-effect model and the QMSP in the fixed-effect model (MSP, OR = 2.97, 95% CI = 1.55–5.70; QMSP, OR = 8.84, 95% CI = 5.22–14.99). The pooled ORs in QMSP were higher than in the MSP. The phenomenon could be attributed tothe specificity and sensitivity of QMSP detecting up to 1/1000 methylated alleles more than the conventional MSP [20]. With the control source, the significant association between *DAPK* promoter methylation and HNSCC was found among the autologous group and the heterogeneous group in the random-effect model (autologous group, OR = 2.71, 95% CI = 1.49–4.93; heterogeneous group, OR = 9.50, 95% CI = 2.98–30.27).The results suggested that the frequency of *DAPK* promoter methylationin the autologous control was higher than the heterogeneous control. This indicated that the *DAPK* promoter methylation might play a crucial role in the pathogenesis of HNSCC.

We also investigated the correlation between the *DAPK* promoter methylation and the clinicopathological features. The results suggested that *DAPK* promoter methylation was significantly correlated with the alcohol status. The drinkers have a 1.85-fold increased *DAPK* methylation frequency compared with the non-drinkers (OR = 1.85, 95% CI = 1.07–3.21). The *DAPK* promoter methylation was not significantly correlated with sex, smoking, and lymph node invasion.

However, the present meta-analysis exhibited some limitations. First, a limited number of articles were included in the meta-analysis for assessing the correlation between *DAPK* promoter methylation and clinicopathological features. Thus, the accurate and reasonable conclusions need to be confirmed in future studies. Second, although the publication bias was not significant according to Egger’s test, some unpublished studies and non-English language studies may contribute to some bias.

In conclusion, the present study found that aberrant methylation of *DAPK* promoter was associated with HNSCC, which suggested that the promoter methylation of *DAPK* plays a crucial role in the development of HNSCC. However, well-designed studies with larger sample size may be performed in order to further confirm the correlation between *DAPK* promoter methylation and HNSCC.

## Supporting information

S1 Checklist PRISMA 2009 checklist. (DOC)

S2 Checklist Meta-analysis-on-genetic-association-studies checklist. (DOCX)

S3 Checklist PLOS One clinical studies checklist. (DOCX)

## References

[1] Siegel R, Ma J, Zou Z, Jemal A. Cancer statistics, 2014. CA Cancer J Clin. 2014; 64(1):9–29. 10.3322/caac.21208 24399786

[2] Leemans CR, Braakhuis BJ, Brakenhoff RH. The molecular biology of head and neck cancer. Nat Rev Cancer. 2011; 11(1):9–22. 10.1038/nrc2982 21160525

[3] Guerrero-Preston R, Michailidi C, Marchionni L, Pickering CR, Frederick MJ, Myers JN, et al Key tumor suppressor genes inactivated by "greater promoter" methylation and somatic mutations in head and neck cancer. Epigenetics. 2014; 9(7):1031–1046. 10.4161/epi.29025 24786473 PMC4143405

[4] Misawa Y, Misawa K, Kanazawa T, Uehara T, Endo S, Mochizuki D, et al Tumor suppressor activity and inactivation of galanin receptor type 2 by aberrant promoter methylation in head and neck cancer. Cancer. 2014; 120(2):205–213. 10.1002/cncr.28411 24122450

[5] Gozuacik D, Kimchi A. DAPk protein family and cancer. Autophagy. 2006; 2(2):74–79. 17139808 10.4161/auto.2.2.2459

[6] Cohen O, Inbal B, Kissil JL, Raveh T, Berissi H, Spivak-Kroizaman T, et al DAP-kinase participates in TNF-alpha- and Fas-induced apoptosis and its function requires the death domain. J Cell Biol. 1999; 146(1):141–148. 10402466 10.1083/jcb.146.1.141 PMC2199731

[7] Velentza AV, Schumacher AM, Weiss C, Egli M, Watterson DM. A protein kinase associated with apoptosis and tumor suppression: structure, activity, and discovery of peptide substrates. J Biol Chem. 2001; 276(42):38956–38965. 10.1074/jbc.M104273200 11483604

[8] Li C, Wang L, Su J, Zhang R, Fu L, Zhou Y. mRNA expression and hypermethylation of tumor suppressor genes apoptosis protease activating factor-1 and death-associated protein kinase in oral squamous cell carcinoma. Oncol Lett. 2013; 6(1):280–286. 10.3892/ol.2013.1353 23946818 PMC3742820

[9] Christoph F, Kempkensteffen C, Weikert S, Kollermann J, Krause H, Miller K, et al Methylation of tumour suppressor genes APAF-1 and DAPK-1 and in vitro effects of demethylating agents in bladder and kidney cancer. Br J Cancer. 2006; 95(12):1701–1707. 10.1038/sj.bjc.6603482 17133271 PMC2360762

[10] Bai J, Zhang X, Hu K, Liu B, Wang H, Li A, et al Silencing DNA methyltransferase 1 (DNMT1) inhibits proliferation, metastasis and invasion in ESCC by suppressing methylation of RASSF1A and DAPK. Oncotarget. 2016. 10.18632/oncotarget.9866 PMC5190084 27286455

[11] Wang W, Wang J, Li Z, Zhu M, Zhang Z, Wang Y, et al Promoter hypermethylation of PTPL1, PTPN6, DAPK, p16 and 5-azacitidine inhibits growth in DLBCL. Oncol Rep. 2016; 35(1):139–146. 10.3892/or.2015.4347 26498513

[12] Niklinska W, Naumnik W, Sulewska A, Kozlowski M, Pankiewicz W, Milewski R. Prognostic significance of DAPK and RASSF1A promoter hypermethylation in non-small cell lung cancer (NSCLC). Folia Histochem Cytobiol. 2009; 47(2):275–280. 10.2478/v10042-009-0091-2 19926549

[13] Liberati A, Altman Dg Fau—Tetzlaff J, Tetzlaff J Fau—Mulrow C, Mulrow C Fau—Gotzsche PC, Gotzsche Pc Fau—Ioannidis JPA, Ioannidis Jp Fau—Clarke M, et al. The PRISMA statement for reporting systematic reviews and meta-analyses of studies that evaluate healthcare interventions: explanation and elaboration. (1756–1833 (Electronic)).

[14] Higgins JP, Thompson SG. Quantifying heterogeneity in a meta-analysis. Stat Med. 2002; 21(11):1539–1558. 10.1002/sim.1186 12111919

[15] Begg CB, Mazumdar M. Operating characteristics of a rank correlation test for publication bias. Biometrics. 1994; 50(4):1088–1101. 7786990

[16] Egger M, Davey Smith G, Schneider M, Minder C. Bias in meta-analysis detected by a simple, graphical test. Bmj. 1997; 315(7109):629–634. 9310563 10.1136/bmj.315.7109.629 PMC2127453

[17] Ogi K, Toyota M, Ohe-Toyota M, Tanaka N, Noguchi M, Sonoda T, et al Aberrant methylation of multiple genes and clinicopathological features in oral squamous cell carcinoma. Clin Cancer Res. 2002; 8(10):3164–3171. 12374684

[18] Inbal B, Cohen O, Polak-Charcon S, Kopolovic J, Vadai E, Eisenbach L, et al DAP kinase links the control of apoptosis to metastasis. Nature. 1997; 390(6656):180–184. 10.1038/36599 9367156

[19] Li C, Wang L, Su J, Zhang R, Fu L, Zhou Y. mRNA expression and hypermethylation of tumor suppressor genes apoptosis protease activating factor-1 and death-associated protein kinase in oral squamous cell carcinoma. Oncology Letters. 2013; 6(1):280–286. 10.3892/ol.2013.1353 23946818 PMC3742820

[20] Fackler MJ, Malone K, Zhang Z, Schilling E, Garrett-Mayer E, Swift-Scanlan T, et al Quantitative multiplex methylation-specific PCR analysis doubles detection of tumor cells in breast ductal fluid. Clin Cancer Res. 2006; 12(11 Pt 1):3306–3310. 16740751 10.1158/1078-0432.CCR-05-2733

[21] Arantes LM, de Carvalho AC, Melendez ME, Centrone CC, Gois-Filho JF, Toporcov TN, et al Validation of methylation markers for diagnosis of oral cavity cancer. Eur J Cancer. 2015; 51(5):632–641. 10.1016/j.ejca.2015.01.060 25686481

[22] Choudhury JH, Ghosh SK. Promoter Hypermethylation Profiling Identifies Subtypes of Head and Neck Cancer with Distinct Viral, Environmental, Genetic and Survival Characteristics. PLoS One. 2015; 10(6):e0129808 10.1371/journal.pone.0129808 26098903 PMC4476679

[23] Rettori MM, de Carvalho AC, Longo AL, de Oliveira CZ, Kowalski LP, Carvalho AL, et al TIMP3 and CCNA1 hypermethylation in HNSCC is associated with an increased incidence of second primary tumors. J Transl Med. 2013; 11:316 10.1186/1479-5876-11-316 24359512 PMC3884019

[24] Liu Y, Zhou ZT, He QB, Jiang WW. DAPK promoter hypermethylation in tissues and body fluids of oral precancer patients. Med Oncol. 2012; 29(2):729–733. 10.1007/s12032-011-9953-5 21516484

[25] Paluszczak J, Misiak P, Wierzbicka M, Wozniak A, Baer-Dubowska W. Frequent hypermethylation of DAPK, RARbeta, MGMT, RASSF1A and FHIT in laryngeal squamous cell carcinomas and adjacent normal mucosa. Oral Oncol. 2011; 47(2):104–107. 10.1016/j.oraloncology.2010.11.006 21147548

[26] Wong Y-K, Lee L-T, Liu C-J. Hypermethylation of MGMT and DAPK gene promoters is associated with tumorigenesis and metastasis in oral squamous cell carcinoma. Journal of Dental Sciences. 2011; 6(3):158–164.

[27] Laytragoon-Lewin N, Chen F, Castro J, Elmberger G, Rutqvist LE, Lewin F, et al DNA content and methylation of p16, DAPK and RASSF1A gene in tumour and distant, normal mucosal tissue of head and neck squamous cell carcinoma patients. Anticancer Res. 2010; 30(11):4643–4648. 21115918

[28] Su PF, Huang WL, Wu HT, Wu CH, Liu TY, Kao SY. p16(INK4A) promoter hypermethylation is associated with invasiveness and prognosis of oral squamous cell carcinoma in an age-dependent manner. Oral Oncol. 2010; 46(10):734–739. 10.1016/j.oraloncology.2010.07.002 20729138

[29] Steinmann K, Sandner A, Schagdarsurengin U, Dammann RH. Frequent promoter hypermethylation of tumor-related genes in head and neck squamous cell carcinoma. Oncol Rep. 2009; 22(6):1519–1526. 19885608 10.3892/or_00000596

[30] De Schutter H, Geeraerts H, Verbeken E, Nuyts S. Promoter methylation of TIMP3 and CDH1 predicts better outcome in head and neck squamous cell carcinoma treated by radiotherapy only. Oncol Rep. 2009; 21(2):507–513. 19148529

[31] Righini CA, de Fraipont F, Timsit JF, Faure C, Brambilla E, Reyt E, et al Tumor-specific methylation in saliva: a promising biomarker for early detection of head and neck cancer recurrence. Clin Cancer Res. 2007; 13(4):1179–1185. 10.1158/1078-0432.CCR-06-2027 17317827

[32] Kong WJ, Zhang S, Guo C, Zhang S, Wang Y, Zhang D. Methylation-associated silencing of death-associated protein kinase gene in laryngeal squamous cell cancer. Laryngoscope. 2005; 115(8):1395–1401. 10.1097/01.MLG.0000166708.23673.3A 16094112

[33] Kulkarni V, Saranath D. Concurrent hypermethylation of multiple regulatory genes in chewing tobacco associated oral squamous cell carcinomas and adjacent normal tissues. Oral Oncol. 2004; 40(2):145–153. 14693237 10.1016/s1368-8375(03)00143-x

[34] Misawa K, Mochizuki D, Imai A, Endo S, Mima M, Misawa Y, et al Prognostic value of aberrant promoter hypermethylation of tumor-related genes in early-stage head and neck cancer. Oncotarget. 2016. 10.18632/oncotarget.8317 PMC5041966 27027429

[35] Pierini S, Jordanov SH, Mitkova AV, Chalakov IJ, Melnicharov MB, Kunev KV, et al Promoter hypermethylation of CDKN2A, MGMT, MLH1, and DAPK genes in laryngeal squamous cell carcinoma and their associations with clinical profiles of the patients. Head Neck. 2014; 36(8):1103–1108. 10.1002/hed.23413 23804521

[36] Supic G, Kozomara R, Brankovic-Magic M, Jovic N, Magic Z. Gene hypermethylation in tumor tissue of advanced oral squamous cell carcinoma patients. Oral Oncol. 2009; 45(12):1051–1057. 10.1016/j.oraloncology.2009.07.007 19665921

